# Putting youth at the centre: co-design of a community-based intervention to improve HIV outcomes among youth in Zimbabwe

**DOI:** 10.12688/wellcomeopenres.17531.2

**Published:** 2022-09-30

**Authors:** Constance RS. Mackworth-Young, Stefanie Dringus, Ethel Dauya, Chido Dziva Chikwari, Constancia Mavodza, Mandikudza Tembo, Aoife Doyle, Grace McHugh, Victoria Simms, Maurice Wedner-Ross, Tsitsi Apollo, Owen Mugurungi, Rashida A. Ferrand, Sarah Bernays

**Affiliations:** 1 Department of Global Health and Development, London School of Hygiene & Tropical Medicine, London, UK; 2 Biomedical Research and Training Institute, Harare, Zimbabwe; 3 MRC International Statistics and Epidemiology Group, London School of Hygiene & Tropical Medicine, London, UK; 4 AIDS and TB Unit, Ministry of Health and Child Care, Harare, Zimbabwe; 5 Department of Clinical Research, London School of Hygiene & Tropical Medicine, London, UK; 6 School of Public Health, University of Sydney, Sydney, Australia

**Keywords:** Adolescents, Youth, HIV care continuum, HIV prevention, sub-Saharan Africa

## Abstract

**Background:**

Youth have disproportionately poor HIV outcomes. We aimed to co-design a community-based intervention with youth to improve HIV outcomes among 16–24 year-olds, to be trialled in Zimbabwe.

**Methods:**

We conducted 90 in-depth interviews with youth, family members, community gatekeepers and healthcare providers to understand the barriers to uptake of existing HIV services. The interviews informed an outline intervention, which was refined through two participatory workshops with youth, and subsequent pilot-testing.

**Results:**

Participants considered existing services inaccessible and unappealing: health facilities were perceived to be for ‘sick people’, centred around HIV and served by judgemental providers. Proposed features of an intervention to overcome these barriers included: i) delivery in a youth-only community space; ii) integration of HIV services with broader health services; iii) non-judgemental skilled healthcare providers; iv) entertainment to encourage attendance; and v) tailored timings and outreach. The intervention framework stands on three core pillars, based on optimizing access (community-based youth-friendly settings); uptake and acceptability (service branding, confidentiality, and social activities); and content and quality (integrated HIV care cascade, high quality products, and trained providers).

**Conclusions:**

Ongoing meaningful youth engagement is critical to designing HIV interventions if access, uptake, and coverage is to be achieved.

## Introduction

**Figure 1.**
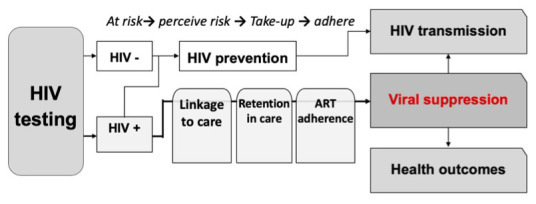
The HIV care cascade. HIV care outcomes are disproportionately worse across the care continuum for youth.

HIV-associated mortality has declined dramatically globally as a result of antiretroviral therapy (ART) in all age groups except among youth ( UNAIDS, 2019). Youth fare disproportionately poorly across each step of the HIV care cascade: the prevalence of undiagnosed HIV infection is higher, coverage of ART is lower, and treatment adherence is poorer ( Figure 1) ( Floyd *et al.*, 2018; Hayes *et al.*, 2019). Together these factors result in worse virological outcomes ( Vogt *et al.*, 2017).

Youth (defined in this paper as those aged 16–24 years) face substantial personal, social and structural barriers to accessing HIV services in health facilities ( Cluver *et al.*, 2018). Community-based HIV care, including through support from community health workers ( Ferrand *et al.*, 2017) or community adolescent treatment supporters ( Mavhu *et al.*, 2020) can improve treatment access and virological outcomes among children and young people. Furthermore, approaches that address the entire cascade of HIV care (HIV testing; linkage to care and ART initiation; and adherence support to improve viral suppression) ( Figure 1) may reduce the attrition that occurs at each step of the care cascade ( Shanaube *et al.*, 2021).

A recent systematic review highlighted the scarcity of evidence to inform effective HIV service delivery models for youth, and the urgent need to design and evaluate interventions outside of health facilities ( Casale *et al.*, 2019). There is general consensus that to design interventions that can effectively tackle the health challenges faced by youth, they must be involved ( Denison *et al.*, 2017; Melles & Ricker, 2018). Larson’s application of Shier’s Pathways to Participation Model ( Shier, 2001) describes the higher levels of participation in the development of interventions: level 3 where children and young people’s views are taken into account; level 4 where they are involved in decision-making processes; and the highest, level 5, where they share responsibility in the development of interventions ( Larsson *et al.*, 2018). However, despite an increasing rhetoric emphasising the importance of youth involvement in research, there continues to be relatively limited adoption of practices which meet these higher levels of youth participation in intervention development ( Larsson *et al.*, 2018). The lack of genuine involvement of youth is in part due to the lack of critical reflection on the implementation of youth co-design, including the opportunities and challenges, and the extent to which it can genuinely enhance intervention design and uptake.

In this paper we contribute to this nascent evidence-base by describing the co-design of a community-based intervention aimed at improving HIV outcomes among youth in Zimbabwe, a country that has experienced a severe and sustained generalised HIV epidemic. In 2020, Zimbabwe’s youth HIV prevalence was 3.8% and 2.1% among 15–19 year old women and men respectively, and 6.4% and 2.8% among 20–24 year old women and men respectively ( ZIMPHIA, 2020).

Our goal for this paper is two-fold. Firstly, we describe the co-design process of the CHIEDZA intervention, with youth as partners, detailing how young people shaped and refined the intervention design. Secondly, we share learning and critical reflection on the co-design process, with the aim of contributing to the development of empirically-informed best practices for youth engagement in the design of youth-focused HIV interventions.

This study influenced the design of a protocol for a cluster-randomised trial which can be seen here ( Dziva Chikwari *et al.*, 2022).

## Methods

### Study design

CHIEDZA stands for community-based interventions to improve HIV outcomes in youth: a cluster randomis ed trial in Zimb abwe. At conceptualisation, the proposed CHIEDZA intervention was defined by three main components, namely: 1) addressing the entire HIV care cascade (HIV testing; linkage to care and ART initiation; and adherence support to improve viral supression); 2) combining HIV prevention and the HIV care cascade; and 3) delivery in a community-based setting. The trial is currently being conducted in 24 communities in three provinces in Zimbabwe (Harare, Mashonaland East and Bulawayo), with the outcome, HIV viral suppression, to be ascertained at population level. 

The CHIEDZA intervention was co-designed with youth in Zimbabwe in three phases conducted over 12 months in 2018. This co-design process was carried out in three of the 24 communities where the CHIEDZA trial would be conducted, purposefully selected to represent the variation across study communities. 

### Phase 1: In-depth Interviews

Ninety in-depth interviews (IDIs) were conducted with seven groups of participants. Youth were recruited by referral from primary care clinics and community-based organizations, presented ( Table 1). Their eligibility was based on whether they fitted into one of the seven categories outlined in Table 1. eligible participants were then purposefully sampled for maximum variation. For example, within the group of youth living with HIV, participants represented a range of ages, gender, mode of HIV acquisition, length since HIV diagnosis, and their level of participation in HIV support services ( Table 1).

**Table 1.** In-depth interview sample.

Type of IDI Respondent	Total Number of Participants	Male	Female	Age Range (years)
CBO representatives	9	3	5	34–53
Healthcare Providers (Facility-Based)	8	1	7	29–40
Healthcare Providers (Community-Based)	7	0	7	34–51
Youth (HIV Negative)	25	12	13	16–25
Youth (HIV Positive)	26	14	12	16–24
Parents and Family Members of Youth	11	2	9	37–58
Community Gate Keepers	4	1	3	39–62
**Total**	90	33	56	16–62

Within the interviews we sought to a) understand the barriers and facilitators youth face in accessing existing services, and b) elicit suggestions for an intervention that would optimise access, uptake, and coverage. Interviews were conducted by trained qualitative researchers using semi-structured topic guides, tailored to each participant group. Interviews were conducted in health facilities within study communities, in either Shona or English depending on the participant’s language preference, and were audio recorded.

Detailed summaries of each interview were written and checked for comprehensiveness against the audio recording. Interview summaries are an effective method of documenting interview content and interviewer reflections, and can serve as an initial stage of analysis ( Rutakumwa *et al.*, 2020). Data collection and analysis were iterative, informing the refinement of sampling approaches, topic guides and the emerging draft intervention. Summaries were manually coded for themes and organised by participant group. Analysis included reflexive memos. This enabled the syntheses of emerging findings and identified areas for further follow-up in subsequent interviews. These analytical documents were shared weekly for discussion among the research team. Detailed data analysis enabled us to identify the key components of what would constitute an accessible and acceptable intervention and formed a working outline of the intervention design.

### Phase 2: Participatory workshops

In the next phase, the outline of the intervention was presented to youth in two participatory workshops, with a total of 45 youth aged 16–24 years. The workshops aimed to create a collaborative decision-making space to refine the components of the intervention and check interpretation of phase 1 data. Specifically, the workshops focused on the detail of the intervention, including what services and commodities the intervention would offer, the mode of delivery, branding, and the look and feel of the space. Activities included brainstorming, ideas, as well as ranking, and discussion exercises. The workshops were facilitated in a mixture of Shona and English (depending on participant preference) by the research team and external facilitators. 

Workshop participants included youth who had participated in phase 1 IDIs and youth who had not previously been involved. The latter group were recruited to extend the range of views and improve generalisability of the intervention design. The first workshop was conducted with youth who had negative or unknown HIV status (n=11 males and 13 females), and the second with a group of HIV positive youth (n=10 males and 11 females). This separation was done to avoid unintentional HIV status disclosure, and to enable more detailed exploration of the lived experience of HIV treatment and care.

Workshops were held in a private room and were audio recorded. Written outputs from the workshops, such as ranking lists, were photographed. Workshop summaries were produced, informed by the audio recordings. After each workshop, detailed summaries of both the workshops and de-brief meetings with the research team were written up for analysis. This enabled further refinement of the key priorities and components of the intervention. A draft manual of operations detailing the pilot intervention was produced.

### Phase 3: Pilot

The intervention was piloted over three months in one of the selected intervention communities. Based on uptake data and feedback from youth clients, the intervention design was then further adapted. The manual of operations was refined and the final version is available on request.

### Ethical approval

Ethical approval was granted by Medical Research Council of Zimbabwe (MRC/A/2266), London School of Hygiene and Tropical Medicine (14652) and Biomedical Research and Training Institute (AP144/2018). All participants provided written informed consent. A waiver for the need of guardian consent was granted for participants aged 16–18 years to enable them to them share their views without censure from guardians.

## Results

### Barriers to youth accessing existing health services

To design the intervention, we first sought to understand whether and why current services were failing to meet the needs of youth. A consistent finding was that existing services (largely facility-based) were both unappealing and inaccessible to youth, translating into low uptake. See Table 2 for quotes.

**Table 2.** Appeal and accessibility factors undermining access to existing health services: quotes.

Appeal barrier 1: Health services were considered for ‘sick people’ and centring on HIV	• “ *For those who are HIV negative, they find it difficult to come and access these services at the* * facility: they don’t think the services are for them*.” (Healthcare provider) • “ *People are given wrong information by peers; that if you test HIV positive you are going to die. So,* * someone will be afraid of getting tested and knowing their status*.” (Young man, HIV-negative, 24 years)
Appeal barrier 2: Fear of judgement, which could spread around the community	• “ *If I visit the clinic, the nurses will know that so and so is now engaging in sexual activities. People* * will say you are promiscuous*” (Young woman, HIV-negative, 23 years). • “ *When a 15 year old goes to the clinic to ask for contraceptives, they may be turned away because* * there is a belief that youth are too young for sex.*” (Family member) • “ *When you tell them your problem, you will hear them say, ‘at your age how come you contracted* * that disease?*’” (Young man living with HIV, 18 years). • “ *Youths need their own place for accessing services where they are not interrupted by adults, * *otherwise, they shy out*” (Community based organisation)
Accessibility barriers	• “ *Even for those youth who are willing to come to the clinic, they don’t have money to access* * services*” (Family member) • “ *If you come at 7 and only get the service at 1, a small service, then that person will never come* * back again*.” (Young man living with HIV, 24 years)

*1) Appeal barrier 1: Existing health services were considered for ‘sick people’ and centered on HIV*

Youth participants considered that health facilities existed to treat ‘sick people’ and were not places for them as they considered themselves a healthy age group. There was a perception that health services which did target youth were almost exclusively focused on HIV. Despite the widespread availability of effective treatment, HIV remained associated with being ill, which clashed with youth perceptions’ of their invulnerability to ill-health. In addition, there was a disconnect for many between their knowledge of risk behaviours for acquiring HIV and their recognition of their own personal risk. This reduced the pertinence of HIV prevention services for them as individuals. However, they were also fearful of receiving an HIV diagnosis, which they considered would “ *be the end of the world*” (young man living with HIV, 18 years), which led to a reluctance to test for HIV. Distancing themselves from HIV services, youth prioritised services which they considered would help them to maintain their health, including contraception, support for managing menstruation, general health information and counselling for relationship problems, preventing substance abuse harms, and mental health. However, participants reported that these services were not widely available, with the majority of youth services focused on HIV treatment. While they were aware of some prevention services which offered contraception, those which were less directly connected with health, such as menstrual hygiene and relationship counselling were the least available.

*2) Appeal barrier 2: Fear of judgement*

Fear of social disapproval was cited as a key barrier to accessing existing health services targeted at youth, as attendance was tantamount to advertising that they were sexually active. Youth feared being judged by adults who were attending health facilities. They were also distrustful of healthcare providers’ assurances of confidentiality. Many assumed that their attendance for HIV testing or prevention might lead to community gossip, which could reveal their sexual behaviour which they had been keeping a secret from the significant adults in their lives. There was also a widespread expectation that as some healthcare providers held judgmental attitudes, youth might be refused services even if they did attend.

*3) Accessibility barriers*

Even if youth participants considered that their perceived need for healthcare out-weighed the services’ lack of appeal, they described substantial logistical and financial barriers that impeded their access. Transport and user fees, which for many were a prohibitive cost, made it difficult for youth to afford to attend the clinic. The options to overcome these financial barriers were limited; even among families that could afford clinic user fees and transport costs, many youths feared asking caregivers for money to access services associated with sexual health. Long waiting times and clinic opening hours that did not align with youth’s schedules and schooling hours were additional barriers to access.

### Using co-design to identify key features of an acceptable intervention

**Figure 2.**
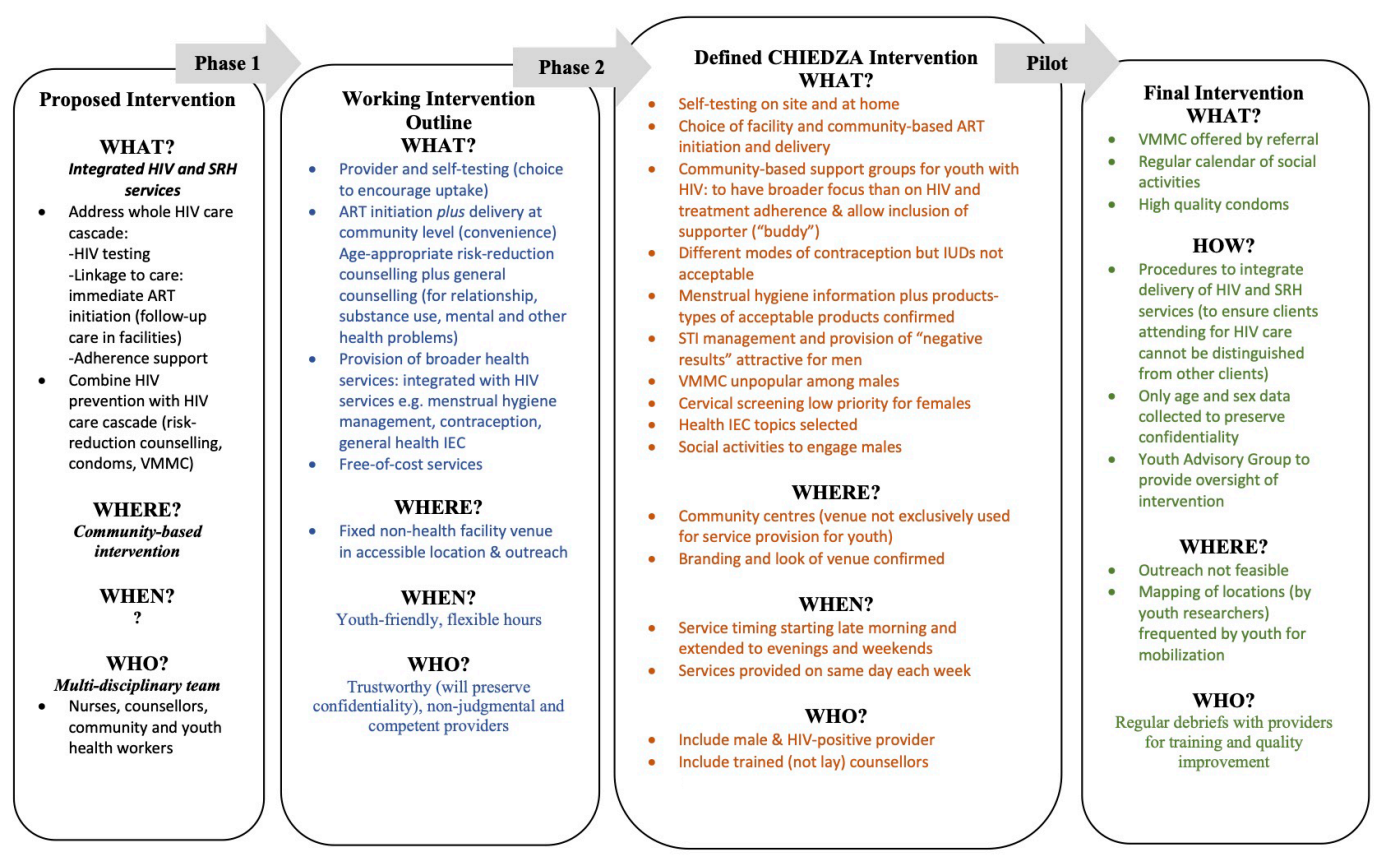
Process of CHIEDZA intervention design, including refinement of the intervention at each phase.

In the IDIs, participants were asked to define the main components of accessible and appealing services. These are described below. This wide range of initial ideas were then further refined through prioritisation exercises during the workshops. Additional adjustments to address feasibility and logistical constraints were identified in the piloting stage. Figure 2 details the iterative process of co-design, and the adaptations and refinements that were made to the intervention at each stage. 

*1) Distinct space for youth*

In the IDIs, many youth and adult participants noted that intervention spaces should be separate from health facilities and distinct from those used by adults, to avoid their attendance being the subject of judgemental gossip within the community. During the workshops, youth participants decided that the best location for the intervention would be in an accessible community venue, such as a community hall. This was preferable to either a health facility or as a standalone mobile intervention, which might attract unwelcome attention due to their existing associations with HIV. Outreach activities including evening events, were proposed, to increase awareness and demand of health interventions especially among young men.

*2) Integration of HIV services with broader health services*

Participants in the IDIs suggested that HIV services should be integrated within the broader health services that were pertinent to the needs of youth. This led to the inclusion of additional services, the specifics of which was refined in the workshops. For example, youth participants identified the provision of menstrual hygiene products as a priority because they would generate demand among young women, while ensuring that the intervention did not mark those attending as being sexually active. Young men particularly desired high-quality condoms. This integrated approach would encourage youth to attend and enable them to take up other services, including HIV testing. To ameliorate the fears around HIV testing identified in the interviews, workshop participants suggested that youth clients’ need not test for HIV the first time they accessed the intervention, rather they could initially develop rapport with and trust in providers when they accessed the intervention for another reason, which would then encourage them to test on a subsequent visit. In the workshops, youth participants emphasised that public branding of the intervention should focus on youth’s general health and wellbeing and not on HIV, to counter potential negative community perceptions. This emphasis would enable youth to justify accessing the intervention according to its pertinence to their age, rather than their attendance being interpreted as indicative of potentially ‘transgressive’ sexual behaviour.

*3) Non-judgemental and qualified healthcare providers*

Across stakeholder groups in the IDIs, the main topic on which views diverged was who should deliver the intervention. Adult participants largely argued that youth involvement in delivery was necessary to increase uptake. However, youth participants envisaged that young people should have a valuable but more limited role, for example having responsibility for mobilising potential clients and supporting client navigation through the intervention. In the workshops, when presented with the option of youth or adult providers, youth participants preferred that the services be provided by experienced and trustworthy adults. They suggested that healthcare providers should be selected on the following attributes: being competent, non-judgemental, approachable, trustworthy and respectful of confidentiality. On this point of disagreement, the views of youth were prioritised over those of adults and so age was not a key criterion in staff recruitment. In the workshops, participants further refined the composition of the provider team to include male healthcare providers and providers living with HIV.

*4) Entertainment and social activities to encourage attendance*

To enable the intervention to have a broader appeal to youth, many youth and adult participants suggested that youth-friendly entertainment should be organised at the sites. This should include music, games such as pool and darts, dancing and fashion. The rationale was that if the intervention could attract youth for whom health was not a priority, particularly young men, this would incidentally expose them to opportunities to access health services.

*5) Tailored timings and outreach for youth*

In the IDIs youth and adult participants mentioned the importance of the intervention being open at times which would fit within the routines of youth. What the ideal timings would be were defined in workshops, with preference for delivery in the afternoon and evenings. This could enable youth, to attend outside of school, work, and house chores, but would not be so late so that they would be forbidden to leave home. Youth suggested that the intervention should be delivered on the same day every week.

***Further refinement of intervention during the pilot***

Youth continued to inform the intervention design throughout the pilot phase. A youth advisory group was established: workshop participants were invited to put their names forward, and the research team purposefully selected 12 youth with diverse experiences and backgrounds. This group met on an ad-hoc basis during the final stages of intervention design and piloting. The group worked closely with a marketing team to brand the intervention and specifically to ensure that outward-facing branding did not focus on HIV or SRH. The youth advisory group were also included as panellists in the interviews to select healthcare providers, so that they could input into the decision-making process regarding the choice of appropriate providers.

Learning from the pilot led to a number of adjustments to the final package of services for the main intervention. Delivering the intervention through evening outreach was not feasible due to recourse constraints. Trained youth researchers identified, areas frequented by youth, and those were targeted for sensitisation activities by youth mobilisers to improve coverage. Instead of providing the same condoms as those delivered within the national program, which had a low uptake during the pilot due to perceptions of them being oily and malodorous, higher quality flavoured and textured condoms, which were branded to focus on pleasure, were provided in the main study. Voluntary medical male circumcision, which had low uptake in the pilot and was reported to discourage young men from attending, was not provided as part of the intervention services within the main study but was instead offered as a referral service. Cervical cancer screening was also removed from the package of intervention services, in line with changes to national guidelines. The final package of services included: HIV testing, linkage to treatment and adherence support, counselling, menstrual hygiene management, branded condoms, family planning, pregnancy testing, testing and treatment of sexually transmitted infections, referral services, and social activities.

## Discussion

**Figure 3.**
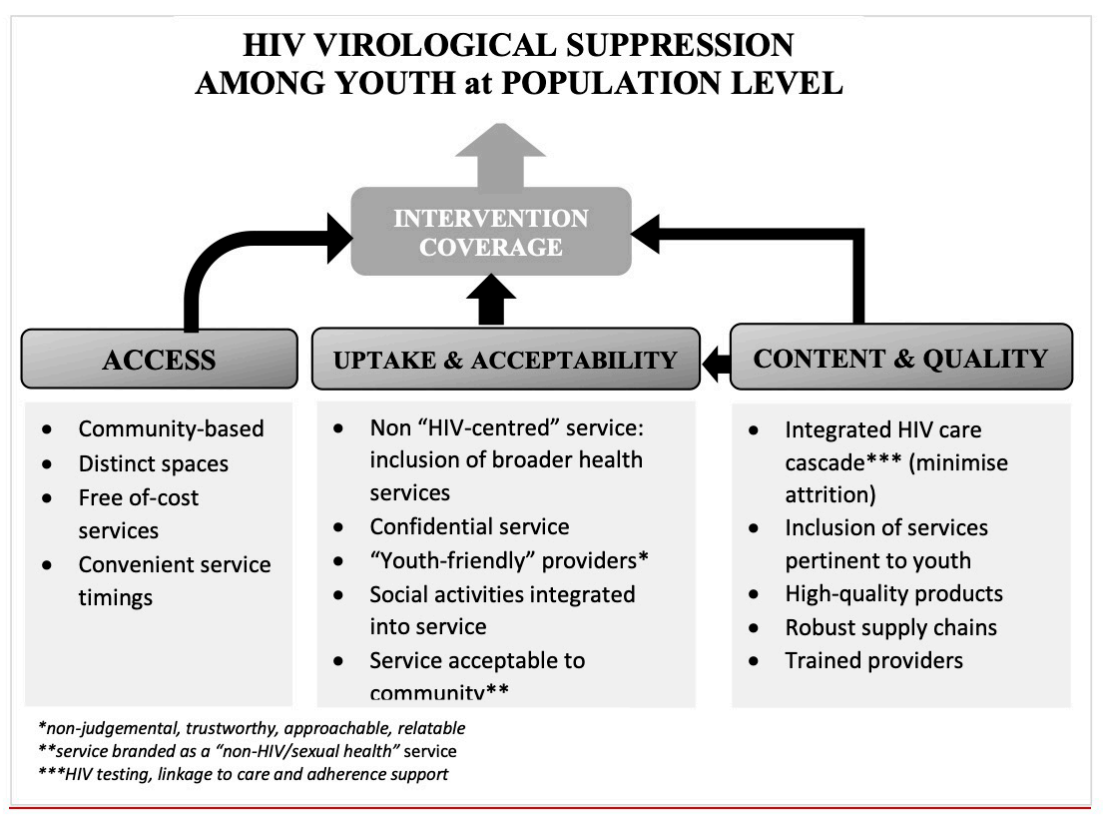
Conceptual framework of CHIEDZA intervention.

The process of co-design with youth facilitated the development and refinement of the CHIEDZA intervention with the aim of improving youth HIV outcomes. This process included listening to a range of views on existing healthcare services from youth and adults. Then, in collaboration with youth, designing the ingredients and formula of an intervention that would maximise access, uptake, and coverage. Through this co-design process the intervention was optimised in respect to three core aspects: i) access: community-based youth-friendly settings; ii) uptake and acceptability: service branding, confidentiality, and social activities; and iii) content and quality: integrated HIV care cascade, high quality products, and trained providers. These three pillars underpinned the CHIEDZA intervention, with the intention that such a design would lead to intervention engagement and coverage, and ultimately improvement of HIV outcomes among youth at a population level ( Figure 3).

The co-design process enabled us to configure the intervention based on the values and preferences of youth. Competence, approachability and a non-judgemental attitude, as well as a commitment to confidentiality, were identified as key attributes of providers, supporting previous research that highlights interpersonal skills, respect, expertise, and communication ( Garrido-Fishbein & Banikya-Leaseburg, 2015). While we had decided *a priori* that the intervention would be delivered in a community-based setting, the exact location (community halls) was defined by youth. Notably, youth highlighted the importance of configuring the intervention so that it was perceived as ‘acceptable’ by adults, because they serve as critical gatekeepers to youth’s access to healthcare. Central to the co-design process was to create an ethos that youth matter and one that celebrates them rather than judges them. This goes beyond current conceptualisations of youth-friendly interventions, where quality of care can be de-prioritised and a lack of appeal impedes access ( Chandra-Mouli *et al.*, 2013; Hoopes *et al.*, 2016). Trust is built through this ethos, supporting uptake and continued engagement with interventions.

To facilitate the adoption of meaningful co-design, Greenhalgh and colleagues demand for the rigorous reporting of approaches ( Greenhalgh *et al.*, 2019), while Larsson and colleagues emphasise the importance of critical reflection on the opportunities and challenges in implementing such approaches in practice ( Larsson *et al.*, 2018). In this vein, we share three key lessons from the co-design process.

Firstly, the co-design process requires considerable time, flexibility, and expectation management. Genuine engagement of youth required the study team and funder to relinquish the need for a pre-determined design of the intervention, and instead be intellectually courageous by trusting in the co-design process. This is a departure from normative practices in which adults’ assumptions predominately shape how we define youth’s needs. Concomitantly, co-design requires there to be explicit and transparent discussions about what is ideal and feasible given resource constraints, with all actors prepared to share power and exercise compromise ( Larsson *et al.*, 2018). Here, youth played an integral role in identifying priority services. Allocating sufficient time for iterative, inclusive, and reflective co-design, in which adaptations could be identified and agreed upon, were critical to the success of the process ( Kime *et al.*, 2013).

Secondly, the co-design process involved listening to multiple voices, identifying consensus and reconciling divergent views. This realignment of power was largely done by recognising the expertise beyond the research team ( Goodyear-Smith *et al.*, 2015; Larsson *et al.*, 2018). Within youth and adult participant groups, power dynamics also played a role: where opinions did diverge, we intentionally privileged the voices of youth where possible. Co-design involves being prepared to be surprised and listening to what youth say they want, rather than using it to affirm expectations. However, it is important to not unwittingly instigate a presumption that youth should, or even want to, be given sole responsibility for designing youth-centred interventions ( Kim & Free, 2008), hence the need for diverse community representation.

Thirdly, co-design needs to include the marginalised and less heard within the youth population ( Marsh *et al.*, 2019). A waiver was applied for and granted to avoid the requirement for parental consent for 16–17 year olds. However, we did not manage to recruit youth from every key groups, for example neither youth with disabilities nor sexual minority youth were represented.

Even with extensive youth participation, by necessity we had to strike a balance between the ideal and the feasible. While the intervention design was unstructured from the beginning, the outcome measure, HIV viral load suppression, was pre-determined. Youth participants were therefore asked to design a pathway to an already defined destination, which was a destination that they had not themselves decided upon. Pragmatism may be necessary to balance openness in intervention design with the ability to leverage the significant funds required for such work.

To facilitate meaningful youth engagement and co-design of HIV interventions, we support Oliveras and colleagues who argue that youth engagement must be adequately resourced with time, training, and funding ( 2018). We additionally suggest that funders should support flexibility in intervention design at the proposal stage, and that ethics committees consider flexibility in requirements of parental informed consent to enable younger adolescents to participate more freely as was done in the design of CHIEDZA ( Goodyear-Smith *et al.*, 2015; Marsh *et al.*, 2019). Through such iterative engagement, we provide lessons learned and key features of co-designing an intervention with youth, that they wanted, and to meet their needs.

The CHIEDZA trial (registered in clinical trials.gov:NCT03719521) is currently being implemented and will be evaluated through an endline population-based survey of youth HIV viral load and process evaluation.

## Data Availability

Data are available upon request. Data underlying our findings cannot be made public for ethical reasons, as they contain information that could compromise the privacy and consent of research participants. Data requests may be sent to the corresponding author including information about how the data will be used and for what purpose, as well as whether specific details of the trial will be included in analytical outputs. LSHTM Data Compass: Community based interventions to improve HIV outcomes in youth: a cluster randomised trial in Zimbabwe (CHIEDZA), https://doi.org/10.17037/DATA.00002718 ( Dziva Chikwari, 2022). This project contains the following extended data: CH01b_CHIEDZA_16_IDI_Consent CH02b_CHIEDZA_HealthWorker_IDI_Consent CH03b_CHIEDZA_HealthcareProvider_IDI_Consent CH04b_CHIEDZA_CommunityOrg_IDI_Consent CH05b_CHIEDZA_FamilyMember_IDI_Consent CH06b_CHIEDZA_Adolescent_HIV_IDI_Consent CH07b_CHIEDZA_CommunityMembers_IDI_Consent CHIEDZA_Adolescent_IDI_TopicGuide CHIEDZA_CommunityGateKeeper_IDI_TopicGuide CHIEDZA_CommunityHealthWorker_IDI_TopicGuide CHIEDZA_FacilityHealthWorker_IDI_TopicGuide CHIEDZA_CommunityOrg_IDI_TopicGuide CHIEDZA_FamilyMember_IDI_TopicGuide Data are available under the terms of the Creative Commons Zero "No rights reserved" data waiver (CC0 1.0 Public domain dedication).
