# Supplementary material for: Putting youth at the centre: co-design of a community-based intervention to improve HIV outcomes among youth in Zimbabwe
Source: Wellcome Open Res. 2022 Sep 30;7:53. Originally published 2022 Feb 14. [Version 2] doi: 10.12688/wellcomeopenres.17531.2 (PMC10804048; doi:10.12688/wellcomeopenres.17531.2)
Supplement: Peer review history

# Reviewer report: Reviewer response for version 2

**DOI:** 10.21956/wellcomeopenres.20409.r71380

**Published:** 2024-01-22

**Authors:** Lario Viljoen

**Affiliations:** 1 Department of Paediatrics and Child Health, Stellenbosch University, Cape Town, South Africa

The authors have done an exceptional job at describing an appropriate intervention for an important and often neglected group. They provide a detailed overview of the steps taken to design an intervention for young people (16 – 24) in Zimbabwe with the overall aim of improving HIV outcomes. This is one of the few times that it is evident that there was a genuine effort to include affected communities (young people) in the co-design of an intervention/package of services.

 I would also like to commend the authors on the use of case summaries and making the argument for using these.

 Overall, the manuscript read well, the methods are systematically described, and the conclusions flow from the results. I only have a few minor comments with one comment for consideration.   

 Minor comments:

- Table 1: Write out CBO
- The authors describe how youth were recruited, but not how other groups of participants were recruited
- Did caregivers include caregivers of children living with and not living with HIV?

For consideration:

- The authors repeatedly emphasise that the service package should include wider services, and that HIV services should be integrated with other services, but the final package of care described seems to include almost exclusively SRH services? Or does ‘referral services’ refer to other health services provided? Perhaps add one sentence to describe other services included.

Is the work clearly and accurately presented and does it cite the current literature?

Yes

If applicable, is the statistical analysis and its interpretation appropriate?

Not applicable

Are all the source data underlying the results available to ensure full reproducibility?

Yes

Is the study design appropriate and is the work technically sound?

Yes

Are the conclusions drawn adequately supported by the results?

Yes

Are sufficient details of methods and analysis provided to allow replication by others?

Yes

Reviewer Expertise:

HIV prevention, community interventions, paediatric TB, stigma

I confirm that I have read this submission and believe that I have an appropriate level of expertise to confirm that it is of an acceptable scientific standard.

# Reviewer report: Reviewer response for version 2

**DOI:** 10.21956/wellcomeopenres.20409.r52579

**Published:** 2022-11-11

**Authors:** Christina A. Laurenzi

**Affiliations:** 1 Institute for Life Course Health Research, Department of Global Health, Faculty of Medicine and Health Sciences, Stellenbosch University, Tygerberg, South Africa

I am satisfied with the authors’ thorough and well-formulated responses and think this paper looks great now.

Is the work clearly and accurately presented and does it cite the current literature?

Yes

If applicable, is the statistical analysis and its interpretation appropriate?

Not applicable

Are all the source data underlying the results available to ensure full reproducibility?

Yes

Is the study design appropriate and is the work technically sound?

Yes

Are the conclusions drawn adequately supported by the results?

Yes

Are sufficient details of methods and analysis provided to allow replication by others?

Yes

Reviewer Expertise:

NA

I confirm that I have read this submission and believe that I have an appropriate level of expertise to confirm that it is of an acceptable scientific standard.

# Reviewer report: Reviewer response for version 1

**DOI:** 10.21956/wellcomeopenres.19383.r49015

**Published:** 2022-03-17

**Authors:** Christina A. Laurenzi

**Affiliations:** 1 Institute for Life Course Health Research, Department of Global Health, Faculty of Medicine and Health Sciences, Stellenbosch University, Tygerberg, South Africa

Thank you for the opportunity to review this exciting work, which shares findings and reflections from the co-design of a youth HIV intervention in Zimbabwe. I have a few comments, questions, and suggestions to strengthen this manuscript, all relatively minor.

**Abstract:**

- I think it is great to share the pillars and sub-themes up front – but I’d recommend a re-formatting for clarity of reading. Perhaps something like:  “The intervention framework stands on three core pillars, based on optimizing access (community-based youth-friendly settings); uptake and acceptability (service branding, confidentiality, and social activities); and content and quality (integrated HIV care cascade, high quality products, and trained providers).”

**Introduction:**

- “Community-based HIV testing strategies may be more accessible”: can the authors say a bit more about this for readers unfamiliar with the subject/area? What do these kinds of strategies entail, and what makes them more accessible? Are they accessible in terms of physically accessible or also accompanied by less stigma?
- The idea of youth involvement, and genuine youth involvement, is raised without describing what this might look like; guiding the design is referred to but it is not clear if this is where it ends (or what that actually is). Can the authors say a bit more about this process and what levels or areas youth ideally would be involved with?
- “This is in part hindered by the paucity of critical reflection on implementation of youth co-design”: this sentence could be simplified and streamlined to read more clearly. Linked to the comment above, I might suggest a brief framing of concepts or ideas linked to youth involvement, youth co-design.
- A general question on age of eligibility – much of the evidence on HIV is grouped by 15-19, 20-24, but I noted youth in this paper refers to ages 16-24. Is there a reason for this discrepancy?
- As you state the goal, I would add the name of the intervention here – it currently reads a bit awkwardly.
- The “goal of the study” final paragraph reflects the premise that the papers’ findings will include learnings and critical reflections on the intervention design process, making this sound like more of a methodology paper. However, in the results above (in abstract) this reads like a more traditional approach to qualitative inquiry and distilling of themes to inform an intervention. I realize these are very closely linked but perhaps you can refine the goal and description of the paper in the closing part of the introduction, to more accurately reflect the data that you will be sharing? If “describing the co-design” is the main aim of this paper, I think this gets a bit lost when you move on to HIV stats and overarching goals.

**Methods:**

- A small point but I would refrain from using “it” on its own—third point of CHIEDZA’s aims. “delivery in a community-based setting” or “delivering the intervention”, etc.
- The methods read very well, and clearly. Can it be clarified what “community spaces” means (where IDIs took place) and whether these were sufficiently private?

**Results:**

- A very minor point, I would keep the order of themes consistent – so “inaccessible and unappealing” as summarised above should appear as themes in that same order.
- Can you say a bit more about the health services which were described as largely not available/which youth prioritised? Are all of these equally hard to obtain (e.g. contraception as hard to access as substance use counseling)? It might be worth adding a bit more detail to this statement to help contextualize for your reader.
- Can the authors add a bit more context on user fees? Are these prohibitive or can most families afford them? This might be contextualized with a small note/description.
- To mirror the first sub-head of Results, maybe the second one (CHIEDZA intervention- co-design) might be cast as “Key features of an acceptable intervention” or something like this? This might help to distinguish the individuals’ data and inputs at this stage from the finalised, emergent intervention as it was manualised.
- This sentence could be clarified: “Outreach including evening events, was proposed, to increase awareness and…” – suggest something like “Outreach activities, including evening events, were proposed to increase awareness and demand…”.
- Really interesting findings on the discrepancy between adult and youth preferences for delivery agents!
- The “continued refinement” header seems to be formatted differently than the others, flagging this for the journal.
- Can the text/size of Figure 2 be made bigger/more legible? This is more a query for the journal.

**Discussion:**

- This section reads very well and the reflections are excellent.

**Minor typos/fixes:** I would encourage the authors to proofread for small punctuation /grammar errors, a few examples include:

- Abstract: “Proposed features of an intervention to overcome these barriers, included” should not have a comma.
- Intro: “age-groups” should be two words, same with “evidence-base”.
- Methods: “Community based” should be hyphenated for consistency.
- Results: “out-weighed” should be “outweighed”.
- Discussion: These sentences should be merged to be grammatically correct: “This process included listening to a range of views on existing healthcare services from youth and adults. Then, in collaboration with youth, designing the ingredients and formula of an intervention that would maximise access, uptake, and coverage.”
- Discussion: sharing a revised version of this sentence here- “However, we did not manage to recruit youth from every key group; for example, neither youth with disabilities nor sexual minority youth were represented.”

Is the work clearly and accurately presented and does it cite the current literature?

Yes

If applicable, is the statistical analysis and its interpretation appropriate?

Not applicable

Are all the source data underlying the results available to ensure full reproducibility?

Yes

Is the study design appropriate and is the work technically sound?

Yes

Are the conclusions drawn adequately supported by the results?

Yes

Are sufficient details of methods and analysis provided to allow replication by others?

Yes

Reviewer Expertise:

HIV, adolescent mental health, participatory engagement, qualitative research, implementation science

I confirm that I have read this submission and believe that I have an appropriate level of expertise to confirm that it is of an acceptable scientific standard, however I have significant reservations, as outlined above.

# Response

**Published:** 2022-09-10

**Authors:** Chido Dziva Chikwari

**Affiliations:** London School of Hygiene & Tropical Medicine, UK

**General**

Reviewer comment 1.1 (General): Thank you for the opportunity to review this exciting work, which shares findings and reflections from the co-design of a youth HIV intervention in Zimbabwe. I have a few comments, questions, and suggestions to strengthen this manuscript, all relatively minor.

Author response 1.1: Thank you for your time and your detailed and thoughtful review of the manuscript.

**Abstract**

Reviewer comment 1.2 (Abstract): I think it is great to share the pillars and sub-themes up front – but I’d recommend a re-formatting for clarity of reading. Perhaps something like:  “The intervention framework stands on three core pillars, based on optimizing access (community-based youth-friendly settings); uptake and acceptability (service branding, confidentiality, and social activities); and content and quality (integrated HIV care cascade, high quality products, and trained providers).”

Author response 1.2: We have reworded, as you suggested:

 “ *The intervention framework stands on three core pillars, based on optimizing access (community-based youth-friendly settings); uptake and acceptability (service branding, confidentiality, and social activities); and content and quality (integrated HIV care cascade, high quality products, and trained providers).” *(page 3)

**Introduction**

Reviewer comment 1.3 (Introduction): “Community-based HIV testing strategies may be more accessible”: can the authors say a bit more about this for readers unfamiliar with the subject/area? What do these kinds of strategies entail, and what makes them more accessible? Are they accessible in terms of physically accessible or also accompanied by less stigma?

Author response 1.3: We have reworded this to read:

 “ *Community-based HIV care, including through support from community health workers (Ferrand et al., 2017) or community adolescent treatment supporters (Mavhu., 2020) can improve treatment access and virological outcomes among children and young people*.” (page 4)

Reviewer comment 1.4 (Introduction): The idea of youth involvement, and genuine youth involvement, is raised without describing what this might look like; guiding the design is referred to but it is not clear if this is where it ends (or what that actually is). Can the authors say a bit more about this process and what levels or areas youth ideally would be involved with?

Author response 1.4: We have made the following edits:

 “ *Larson’s application of Shier’s Pathways to Participation Model (Shier, 2001) describes the higher levels of participation in the development of interventions: level 3 where children and young people’s views are taken into account; level 4 where they are involved in decision-making processes; and the highest, level 5, where they share responsibility in the development of interventions (Larsson et al., 2018). However, despite an increasing  rhetoric emphasising the importance of youth involvement in research, there continues to be relatively limited adoption of practices which meet these higher levels of youth participation in intervention development (Larsson et al., 2018).” *(page 4-5)

Reviewer comment 1.5 (Introduction): “This is in part hindered by the paucity of critical reflection on implementation of youth co-design”: this sentence could be simplified and streamlined to read more clearly. Linked to the comment above, I might suggest a brief framing of concepts or ideas linked to youth involvement, youth co-design.

Author response 1.5: We have reworded that sentence to read:

 “ *The lack of genuine involvement of youth is in part due to the lack of critical reflection on the implementation of youth co-design, including the opportunities and challenges, and the extent to which it can genuinely enhance intervention design and uptake.” *(page 5)

 We have also added some details to conceptually frame youth involvement in development of interventions:

 “ *Larson’s application of Shier’s Pathways to Participation Model* *(Shier, 2001)* *describes the higher levels of participation in the development of interventions: level 3 where children and young people’s views are taken into account; level 4 where they are involved in decision-making processes; and the highest, level 5, where they share responsibility in the development of interventions* *(Larsson et al., 2018)*.” (pages 4-5)

Reviewer comment 1.6 (Introduction): A general question on age of eligibility – much of the evidence on HIV is grouped by 15-19, 20-24, but I noted youth in this paper refers to ages 16-24. Is there a reason for this discrepancy?

Author response 1.6: To enhance the accessibility of the intention to young people it was important that young people could decide for themselves whether they wanted to participate, without having to rely on guardian consent. The Medical Research Council for Zimbabwe granted a waiver of consent for those aged 16 years and older, but not younger children. This meant that we could not include young people aged 15 years. Hence, the intervention was targeted for 16-24 year-olds.

Reviewer comment 1.7 (Introduction): As you state the goal, I would add the name of the intervention here – it currently reads a bit awkwardly.

Author response 1.7: We have reworded that whole paragraph in response to the comment below, including adding the name of the intervention (see response to comment 1.8 below).

Reviewer comment 1.8 (Introduction): The “goal of the study” final paragraph reflects the premise that the papers’ findings will include learnings and critical reflections on the intervention design process, making this sound like more of a methodology paper. However, in the results above (in abstract) this reads like a more traditional approach to qualitative inquiry and distilling of themes to inform an intervention. I realize these are very closely linked but perhaps you can refine the goal and description of the paper in the closing part of the introduction, to more accurately reflect the data that you will be sharing? If “describing the co-design” is the main aim of this paper, I think this gets a bit lost when you move on to HIV stats and overarching goals.

Author response 1.8: We have revised the final paragraph of the introduction to read:

 “ *Our goal for this paper is two-fold. Firstly, we describe the co-design process of the CHIEDZA intervention, with youth as partners, detailing how young people shaped and refined the intervention design. Secondly, we share learning and critical reflection on the co-design process, with the aim of contributing to the development of empirically-informed best practices for youth engagement in the design of youth-focused HIV interventions.”*(page 5)

**Methods**

Reviewer comment 1.9 (Methods): A small point but I would refrain from using “it” on its own—third point of CHIEDZA’s aims. “delivery in a community-based setting” or “delivering the intervention”, etc.

Author response 1.9: We have reworded it to read:

 “ *delivery in a community-based setting*” (page 6)

Reviewer comment 1.10 (Methods): The methods read very well, and clearly. Can it be clarified what “community spaces” means (where IDIs took place) and whether these were sufficiently private?

Author response 1.10: We have reworded this sentence to read:

 “ *Interviews were conducted in health facilities within study communities, in either Shona or English depending on the participant’s language preference, and were audio recorded.” *(page 6)

**Results**

Reviewer comment 1.11 (Results): A very minor point, I would keep the order of themes consistent – so “inaccessible and unappealing” as summarised above should appear as themes in that same order.

Author response 1.11: We have changed the wording order to reflect the order that the themes are presented in:

 “ *A consistent finding was that existing services (largely facility-based) were both unappealing and inaccessible to youth, translating into low uptake*.” (page 9)

Reviewer comment 1.12 (Results): Can you say a bit more about the health services which were described as largely not available/which youth prioritised? Are all of these equally hard to obtain (e.g. contraception as hard to access as substance use counselling)? It might be worth adding a bit more detail to this statement to help contextualize for your reader.

Author response 1.12: We have added more detail on this point:

 “ *Distancing themselves from HIV services, youth prioritised services which they considered would help them to maintain their health, including contraception, support for managing menstruation, general health information and counselling for relationship problems, preventing substance abuse harms, and mental health. However, participants reported that these services were not widely available, with the majority of youth services focused on treatment. While they were aware of some prevention services which offered contraception, those which were less directly connected with health, such as menstrual hygiene and relationship counselling were the least available.” *(pages 9-10)

Reviewer comment 1.13 (Results): Can the authors add a bit more context on user fees? Are these prohibitive or can most families afford them? This might be contextualized with a small note/description.

Author response 1.13: We have added detail on this:

 “ *Transport and user fees, which for many were a prohibitive cost, made it difficult for youth to afford to attend the clinic*.” (page 10)

Reviewer comment 1.14 (Results): To mirror the first sub-head of Results, maybe the second one (CHIEDZA intervention- co-design) might be cast as “Key features of an acceptable intervention” or something like this? This might help to distinguish the individuals’ data and inputs at this stage from the finalised, emergent intervention as it was manualised.

Author response 1.14: We have changed the sub-heading to read:

 “ *Using co-design to identify key features of an acceptable intervention*” (page 10)

Reviewer comment 1.15 (Results): This sentence could be clarified: “Outreach including evening events, was proposed, to increase awareness and…” – suggest something like “Outreach activities, including evening events, were proposed to increase awareness and demand…”.

Author response 1.15: We have changed the wording, as suggested, to read:

 “ *Outreach activities, including evening events, were proposed, to increase awareness and demand of health interventions especially among young men.” *(page 11)

Reviewer comment 1.16 (Results): Really interesting findings on the discrepancy between adult and youth preferences for delivery agents!

Author response 1.16: Thank you for this comment.

Reviewer comment 1.17 (Results): The “continued refinement” header seems to be formatted differently than the others, flagging this for the journal.

Author response 1.17: We have changed the formatting of this heading and will check with the journal when published.

Reviewer comment 1.18 (Results): Can the text/size of Figure 2 be made bigger/more legible? This is more a query for the journal.

Author response 1.18: Thank you for this suggestion: we will discuss it with the journal.

**Discussion**

Reviewer comment 1.19 (Discussion): This section reads very well and the reflections are excellent.

Author response 1.19: Thank you for this comment.

**Typos**

Reviewer comment 1.20 (Typos): I would encourage the authors to proofread for small punctuation /grammar errors, a few examples include: Abstract: “Proposed features of an intervention to overcome these barriers, included” should not have a comma.

Author response 1.20: Thank you for highlighting these punctuation/grammar errors. We have done a thorough proofread of the paper and made edits throughout accordingly. We have removed the comma.

Reviewer comment 1.21 (Typos): Intro: “age-groups” should be two words, same with “evidence-base”.

Author response 1.21: We have removed the hyphens from these words.

Reviewer comment 1.22 (Typos): Methods: “Community based” should be hyphenated for consistency.

Author response 1.22: We have hyphenated “community-based”.

Reviewer comment 1.23 (Typos): Results: “out-weighed” should be “outweighed”.

Author response 1.23: We have change to “outweighed”.

Reviewer comment 1.24 (Typos): Discussion: These sentences should be merged to be grammatically correct: “This process included listening to a range of views on existing healthcare services from youth and adults. Then, in collaboration with youth, designing the ingredients and formula of an intervention that would maximise access, uptake, and coverage.”

Author response 1.24: We have edited the wording to read:

 “ *This process included listening to a range of views on existing healthcare services from youth and adults, and then co-designing with youth the ingredients and formula of an intervention that would maximise access, uptake, and coverage*.” (page 14)

Reviewer comment 1.25 (Typos): Discussion: sharing a revised version of this sentence here- “However, we did not manage to recruit youth from every key group; for example, neither youth with disabilities nor sexual minority youth were represented.”

Author response 1.25: We have revised the wording, as suggested:

 “ *However, we did not manage to recruit youth from every key group; for example, neither youth with disabilities nor sexual minority youth were represented*.” (page 16)
